# Commentary: Efficacy and safety of intravenous monoclonal antibodies in patients with moderate-to-severe active Graves’ ophthalmopathy: a systematic review and meta-analysis

**DOI:** 10.3389/fendo.2024.1394616

**Published:** 2024-05-28

**Authors:** Lingxiao Li, Jihong Wu

**Affiliations:** ^1^ Department of Critical Care Medicine, Wuwei Liangzhou Hospital, Wuwei, Gansu, China; ^2^ Department of Endocrinology, Wuwei Liangzhou Hospital, Wuwei, Gansu, China

**Keywords:** Graves’ ophthalmopathy, monoclonal antibodies, efficacy, meta-analysis, comment

The therapeutic landscape for Graves’ ophthalmopathy (GO) is undergoing significant change, underscored by the pivotal role of intravenous glucocorticoids (GCs) and the emerging efficacy of monoclonal antibodies (mAbs). We recently read an article published in Frontiers in endocrinology by Hu et al. The study compares the results of rituximab, tocilizumab, and teprotumumab in the treatment of moderate to severe GO, providing a nuanced analysis of their relative efficacy and safety profiles (1). We congratulate the authors on a very comprehensive work. However, to further improve the quality and readability of the article, we believe there are several points that could enhance the validity of these findings.

First, the methodological rigor of including both randomized controlled trials and observational studies provides a comprehensive view of the therapeutic potential of mAbs. However, the inclusion of observational studies, while expanding the data set, introduces variability that could potentially bias comparative efficacy results. In addition, the reliance on clinical activity score as the primary measure, while standard, may not fully capture the multifaceted outcomes relevant to patient quality of life.

Second, the nuanced analysis comparing rituximab, tocilizumab, and teprotumumab reveals a complex hierarchy of efficacy and tolerability. While monoclonal antibodies are ushering in a new era of GO treatment, their diverse targets and mechanisms present a labyrinthine picture of therapeutic choices. The differential effects of tocilizumab and teprotumumab on proptosis and diplopia, contrasted with the variable performance of rituximab, underscore the need for personalized treatment strategies tailored to individual patient profiles and disease manifestations.

Third, the discussion of safety profiles is paramount, especially in light of the serious concerns surrounding high-dose GC therapy. The mild to moderate adverse events associated with mAbs, contrasted with the severe, sometimes fatal, hepatotoxicity associated with GCs, is driving a shift toward these novel agents. However, the potential for serious complications, such as opportunistic infections with tocilizumab (2), requires vigilant monitoring and judicious clinical decision-making.

Fourth, the search for optimal dosing and a deeper understanding of the mechanisms of action of mAbs remains unfulfilled. This gap highlights the urgent need for further research, including mechanistic studies and tailored therapeutic regimens (3). In addition, the exploration of mAbs in the treatment of GO promises to reevaluate treatment paradigms and potentially revolutionize patient care for this debilitating disease.

Finally, the comparative analysis of monoclonal antibodies in the treatment of GO opens new avenues for intervention, promising improved efficacy with a more favorable safety profile than traditional glucocorticoids. However, the path from empirical evidence to clinical practice is fraught with unanswered questions and the need for meticulous patient-centered research. As we stand on the cusp of therapeutic innovation, the integration of mAbs into the GO treatment armamentarium must be approached with caution, recognizing the diversity of patient responses and the complexity of autoimmune pathogenesis.

## Author contributions

LL: Formal analysis, Investigation, Writing – original draft. JW: Conceptualization, Methodology, Supervision, Validation, Visualization, Writing – review & editing.
